# Lumasiran for primary hyperoxaluria type 1: What we have learned?

**DOI:** 10.3389/fped.2022.1052625

**Published:** 2023-01-10

**Authors:** Xuan Gang, Fei Liu, Jianhua Mao

**Affiliations:** Department of Nephrology, The Children’s Hospital, Zhejiang University School of Medicine, National Clinical Research Center for Child Health, Hangzhou, China

**Keywords:** lumasiran, primary hyperoxaluria type 1 (PH1), RNA interference (RNAi), pediatrics, targeted therapy

## Abstract

Primary hyperoxaluria type 1 (PH1) is a rare autosomal recessive genetic disorder caused by mutations in the AGXT gene. The hepatic peroxisomal enzyme alanine glyoxylate aminotransferase (AGT) defects encoded by the AGXT gene increase oxalate production, resulting in nephrocalcinosis, nephrolithiasis, chronic kidney disease, and kidney failure. Traditional pharmacological treatments for PH1 are limited. At present, the treatment direction of PH1 is mainly targeted therapy which refer to a method that targeting the liver to block the pathway of the production of oxalate. Lumasiran (OxlumoTM, developed by Alnylam Pharmaceuticals), an investigational RNA interference (RNAi) therapeutic agent, is the first drug approved for the treatment of PH1, which was officially approved by the US Food and Drug Administration and the European Union in November 2020. It is also the only drug that has been shown to decrease harmful oxalate. Currently, there are 5 keys completed and ongoing clinical trials of lumasiran in PH1. Through the three phase III trials that completed the primary analysis period, lumasiran has been shown to be effective in reducing oxalate levels in urine and plasma in different age groups, such as children, adults, and patients with advanced kidney disease, including those on hemodialysis. In addition to clinical trials, cases of lumasiran treatment for PH1 have been reported in small infants, twin infants, and children diagnosed with PH1 after kidney transplantation. These reports confirm the effectiveness and safety of lumasiran. All adverse events were of mild to moderate severity, with the most common being mild, transient injection-site reactions. No deaths or severe adverse events were reported. This article reviews PH1 and lumasiran which is the only approved therapeutic drug, and provide new options and hope for the treatment of PH1.

## Introduction

Primary hyperoxaluria (PH) is a rare autosomal recessive disorder characterized by high endogenous oxalate synthesis and secretion with an estimated prevalence of 1–3 cases per million people (1). In 20%–50% of cases, severe renal insufficiency, or relapses after kidney transplant, occur before diagnosis (2). Increasing oxalate concentration leads to the crystallization of calcium oxalate (CaOx), which causes inflammation and tissue fibrosis, leading to urolithiasis, nephrocalcinosis, and progressive renal damage, eventually leading to chronic kidney disease and end-stage renal disease (ESRD), which requiring renal replacement therapy (1, 3, 4). Under ESRD conditions, a significant decrease in oxalate filtration results in an increase in plasma oxalate (POx) levels, leading to further accumulation of calcium oxalate in the kidney and extrarenal tissues such as skin, bone, heart, retina, and the central nervous system (1, 3, 4). Primary hyperoxaluria can be classified into three types according to different affected genes. Primary hyperoxaluria type 1 is caused by mutations in the AGXT-gene, which encodes alanine-glyoxylate aminotransferase, a pyridoxal 5′ -phosphate-dependent enzyme (5). The mutant gene of primary hyperoxaluria type 2 is GRHPR-gene, which encodes glyoxylate reductase/hydroxypyruvate reductase, and primary hyperoxaluria type 3 is caused by mutations in the HOGA1-gene, which encodes the enzyme 4-hydroxy-2-ketoglutarate aldolase (5). In the above three types of primary hyperoxaluria, type I is the most common and severe, which accounting for 80% of all patients. Almost all patients with PH1 progress to ESRD, which is diagnosed at the median age of 24 years (6). PH1 is estimated to account for 1%–2% of pediatric ESRD population in registries from USA, UK, and Japan (7). Most patients with PH1 present in childhood or early adolescence, and the natural course of untreated PH1 is a progressive decline in renal function, often accompanied by symptomatic nephrolithiasis.

Targeted therapy has always been a new direction of the treatment of PH1, including chaperone therapy, substrate reduction therapy, intestinal oxalate degradation, and so on. Lumasiran, which will be introduced in this paper, is one of the therapeutic drugs in substrate reduction therapy, that reduces the production of glyoxylate and pathological oxalate through inhibiting glycolate oxidase by silencing the HAO1 gene encoding glycolate oxidase (8). Lumasiran received its first approval for the treatment of PH1 in all age groups in the EU on the November 2020 and was approved in the USA for the treatment of adult and children in the same month (9). In recent years, clinical trials of lumasiran in the treatment of PH1 have been completed successively, and more and more cases of lumasiran in the treatment of PH1 have been reported. All these suggest that lumasiran has good efficacy in the treatment of PH1 with little side effects. Lumasiran provides new direction and hope for the treatment of PH1. We discuss the mechanism action of lumasiran in the treatment of PH1, clinical trials and case reports involving efficacy, and observed side effects, focusing on the experience to date in the use of lumasiran for PH1.

## Pathogenesis of primary hyperoxaluria type 1

**Figure 1:**
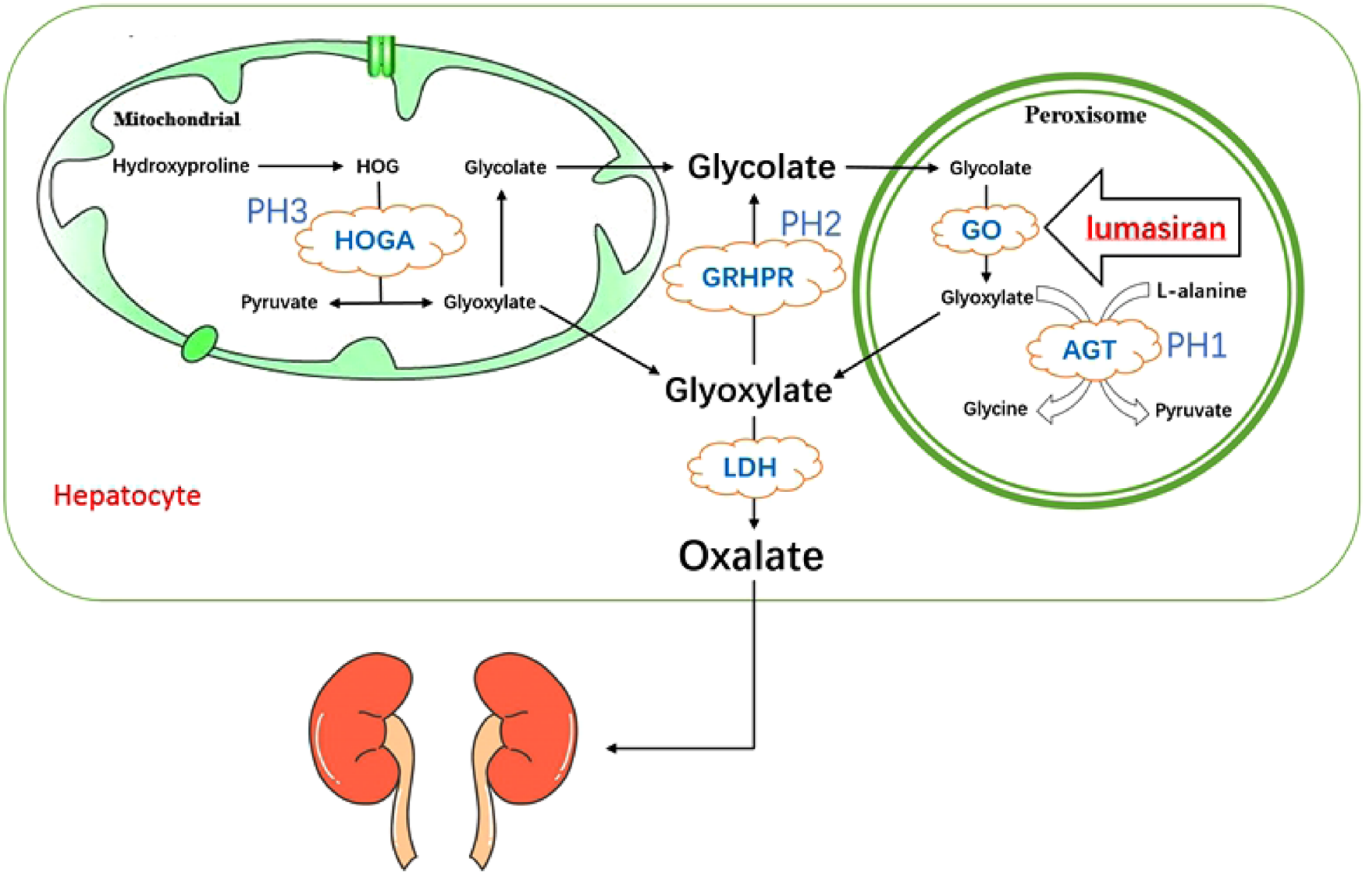
Pathogenesis of primary hyperoxaluria type 1 and the mechanism action of lumasiran. HOGA, 4-hydroxy-2-oxoglutarate aldolase; GRHPR, glyoxylate reductase/hydroxypyruvate reductase; AGT, alanine:glyoxylate aminotransferase; GO, glycolate oxidase; LDH, lactate dehydrogenase.

The occurrence of primary hyperoxaluria is mainly due to the increased production of endogenous oxalic acid excretion. Oxalate is a metabolic end product excreted by urine, and the kidneys keep plasma oxalate levels within normal limits by removing excess oxalate. The removal of oxalate is mainly dependent on the kidney, and synthesis is performed by its main precursor glyoxylate in the liver. In mitochondria, hydroxyproline derived by collagen turnover and metabolism of animal proteins is converted to 4-hydroxy-2-oxoglutarate (HOG), which then converted into glyoxylate and pyruvate by 4-hydroxy-2-ketoglutarate aldolase (HOGA1), as one pathway for glyoxylate production (6). Glyoxylate in mitochondria is converted by glyoxylate reductase/hydroxypyruvate reductase (GRHPR) into glycolate, which goes into peroxisome along with the glycolate which produced by the metabolism of vegetables and fruits, where glycolate is oxidized into glyoxylate by glycolate oxidase (GO), and this is the other pathway for glyoxylate production (6). Glyoxylate are transaminated in peroxisome by alanine-glyoxylate aminotransferase (AGT) to form pyruvate and glycine, along with L-alanine (6). The above two glyoxylate metabolic pathways in mitochondria and peroxidase can ensure that glyoxylate is further metabolized. All glyoxylate that comes from the mitochondria and peroxidase and enters into the cytosol is converted to oxalate by lactate dehydrogenase (LDH) or can also be converted by glyoxylate reductase/hydroxypyruvate reductase (GRHPR) to glycolate, which then enter into the peroxisome to complete the above cycle and to avoid the accumulation of glyoxylate (6). When the enzymes related to glyoxylate metabolism appear deficient, glyoxylate accumulates in the cytosol and will be converted into a large amount of oxalate by lactate dehydrogenase (LDH) (6). Therefore, the underlying cause of increased oxalate production is the deficiency of hepatic glyoxylate metabolizing enzymes (1) (Figure 1).

Key metabolic enzymes in the above metabolic process include alanine-glyoxylate aminotransferase (AGT), 4-hydroxy-2-ketoglutarate aldolase (HOGA1), glyoxylate reductase/hydroxypyruvate reductase (GRHPR). These enzymes are encoded by different genes, mutations in different genes will lead to the deficiency of different enzymes, resulting in different types of primary hyperoxaluria. Among them, alanine-glyoxylate aminotransferase (AGT) is encoded by the AGXT gene, and the mutation of this gene is the genetic basis of PH1. Most of the current studies on PH1 gene mutations are from Western Europe, North Africa, and the Middle East. 52% of the patients have Western European ancestry, and 43% of the patients were originally from North Africa or the Middle East (10). So far, more than 190 mutations have been identified in the AGXT gene (11), among which the three most common were p.G170R, c.33dupC and p.I244T, accounting for approximately 30%, 11% and 6% of the AGXT mutant alleles respectively in European population (2). However, the epidemiology, characteristics, and outcomes of AGXT genotypes in Asia, especially in China, remain unclear. In a single center study and systematic review of primary hyperoxaluria type 1 gene in Chinese, p.Gly41Trp and p.Leu33Met were the first reported mutations in AGXT, while G170R, I244T and F152I, the three most common AGXT mutations mentioned in other reports, were extremely rare in Chinese population (12). It should be noted that due to the small sample size of this single-center study and the limited reporting on PH1 in Chinese population, some variants that are also common in other populations were not identified in the Chinese population.

## Treatment of primary hyperoxaluria type 1

The therapeutic options available for PH1 are poor, most of which are symptomatic treatment, and the treatment effect can only delay the occurrence of ESRD. Therefore, the only confirmed and effective treatment for PH1 is dual liver-kidney transplantation, which can effectively treat metabolic defects, but may have long-term risks, including the need for lifelong immunosuppression (13). The therapeutic strategy for PH1 can be summarized as preserving renal function by preventing CaOx formation or deposition (13). There are traditional approaches that are already in use and new therapies that are being investigated, among which targeted therapy based on disease pathogenesis has become a new field of research for PH1 treatment.

### Traditional treatment

One conservative treatment strategy in the early stages is hyperhydration, which increases daily fluid intake (over 3 L/m^2^ body surface area per day) (14). For special populations, such as infants and children, gastrostomy tubes can be used to ensure adequate hydration day and night, such as fluid loss (fever, vomiting, diarrhea) or surgery patients, intravenous fluids can be given in advance (14). Dietary restrictions are thought to have a limited impact on PH1 patients in the past (4), but a study showed a possible benefit from a low-oxalate diet (15), with vitamin C (which is directly converted to oxalate) (13) and oxalate-rich foods (such as spinach, chocolate, rhubarb, sweet potatoes, cocoa, nuts, and tea) as the main foods to restrict (4, 13, 15). Traditional treatments also include the use of crystallization inhibitors, such as citrate, which increase the solubility of CaOx in urine by forming a complex with calcium, reducing calcium and oxalate precipitation, and thereby reducing CaOx supersaturation (4, 13). Traditional treatment is symptomatic rather than etiological.

### Targeted therapy

The future therapeutic strategies for PH1 are mainly targeted therapies, derived from the molecular basis of PH1, which aims to restore AGT expression, reduce glyoxylate synthesis, improve AGT folding efficiency, and use a functional enzyme that rescues the liver’s glyoxylate detoxification capacity (13). At present, targeted therapies that have passed clinical trials or are under study include chaperone therapy, substrate reduction therapy, intestinal oxalate degradation, and so on.

Chaperone therapy is a small-molecule therapy capable of restoring a functional enzyme conformation change resulting from the most common AGT mutations in PH1, ultimately presenting an overall instability of the protein (6). One of the targeted therapeutic agents that has been used clinically to restore AGT activity in chaperone therapy was supraphysiological doses of vitamin B6, usually administered as pyridoxine hydrochloride (16). Pyridoxine, a prosthetic group for AGT, as well as a molecular chaperone, can promote correct folding of the AGT protein, increase expression, catalytic activity and peroxisomal import of AGT (16, 17). In a PH1 cellular model, pyridoxine 5′-phosphate (PLP), the active ingredient of pyridoxine, was shown to shift the conformation equilibrium to a more stable AGT conformation (18). Two common mutations on the G170R and F152I sequences resulted in a misphenotype of AGT peroxisome to mitochondria, and decreased AGT activity *in vitro* studies (18). The role of vitamin B6 result in successful peroxisomal re-routing of mutant AGT protein, so as to achieve the target effect of restoring AGT activity. This treatment has not been confirmed in reliable clinical trials, but according to the available retrospective case series and studies, only about 30%–50% of patients with PH1 respond to supraphysiological doses of pyridoxine, and some susceptible genotypes exist in this subset (including c.508G > A (p.Gly170Arg), c.454T > A (p.Phe152Ile) and c.121G > A (p.Gly41Arg) mutations) (1). Therefore, this therapy is not effective for all patients with PH1.

In addition to chaperone therapy, another clinically tested therapeutic strategy is substrate reduction, which targets key enzymes in the oxalate metabolic pathway and requires that inhibition of the target must lead to minimal off-target effects (6). There are now three potential targets to treat PH1, which are GO (glycolate oxidase), LDHA (the major hepatic form of LDH), HYPDH (hydroxyproline dehydrogenase), targeted either by enzyme active site inhibition or RNA interference (19). At present, most of the drugs for targeted therapy using enzyme active site inhibition against the above three targets have been proved effective in animal experiments, lack of relevant completed clinical trials. In a genetically modified mice with GO deficiency and only high urine glycolate levels, a GO inhibitor, 4-carboxy-5-[(4-chlorophenyl)sulfanyl]-1,2,3-thiadiazole (CCPST), was shown to be effective in reducing oxalate production, suggesting that GO could be used as an efficient target for substrate reduction therapy in PH1 (20). However, the above drug targeting the inhibition of this enzyme active site have not been made into clinical trials. As the major hepatic form of LDH, LDHA catalyses conversion from glyoxylate into oxalate in the cytosol of hepatocytes as a final step in oxalate generation (21), so it is also considered as another target. Although LDH plays an important role in the interconversion of pyruvate and lactate in tissues, according to relevant reports, LDH-deficient patients do not exhibit any liver-specific phenotype (3), which is also considered as the basis for the safety of this target. In animal models of PH1 and PH2 and mouse models of chemically induced hyperoxaluria, hepatic LDH has also been shown to be an effective target (22), notably, the results of LDHA silencing in a PH2 mouse model were less convincing (23). Stiripentol (Diacomit) has now been shown to be a potent inhibitor of LDH and has been shown to reduce urine oxalate in cell cultures and animal models, whereas the drug was originally used as an antiepileptic agent for Dravet syndrome (6). But Stiripentol did not reduce plasma oxalate levels in dialysis dependent PH1 patients (24), according to a case report, which we conclude that does not have a good effect on every type of PH1 patients particularly showing a severe phenotype. As the first step in catalyzing hydroxyproline decomposition, hydroxyproline dehydrogenase becomes the third target because of its deficiency leading to no other significant pathological sign (13). Following to the characteristics of the enzyme, there are three substrate analogs that can be used as inhibitors, but of which have not entered the clinical trial stage (25). Therefore, this target has not been feasible and safe in clinical practice.

In addition to the above methods of inhibiting enzyme active sites, another method of substrate reduction is RNA interference, which is a natural means of silencing gene expression mediated by small interfering RNA (siRNA) (26). Small interfering RNA (siRNA), which is initiated by double-stranded RNA (dsRNA) *in vivo* and sliced by ribonuclease III (19), binds to a cytoplasmic protein complex (RNA-induced silencing complex) to prevent translation into the corresponding protein (17). Since 2004, this innovative technology has been widely used in drug development programs and entered clinical trials (3). The targets of substrate reduction therapy by iRNA are also focused on GO, LDHA and HYPDH. The targets of substrate reduction therapy by iRNA method are also focused on GO, LDHA and HYPDH, and positive results have been achieved in animal models. Among the three targets mentioned above, GO was the first to be developed, which is encoded by the HAO1 gene and catalyzes the oxidation of glycolate to glyoxylate mainly in liver peroxisomes and has been shown to be a good drug target to reduce oxalate formation in animal models (13). Frishberg Y et al. first introduced children who has asymptomatic glycolic aciduria preceded the pre-clinical trials and has mutations in HAO1 encoding GO (27). The results indicated that the expression of the deficient splicing variant of glycolate oxidase was the cause of isolated asymptomatic glycolic aciduria (27). These studies provide potential for the development of new methods for the treatment of primary hyperoxaluria with substrate reduction. Using data from these animal models, Lumasiran, a drug based on ALNGO1 conjugated to N-acetyl galactosamine (3), targets the mRNA of GO to prevent the conversion of glycolate to glyoxylate, thereby reducing oxalate (17). This article will focus on Lumasiran. Nedosiran (DCR-PHXC), an iRNA drug targeting LDHA, which reduces the cytosolic conversion of glyoxylate to oxalate by silencing LDHA (4), has been demonstrated in animal models and is currently undergoing phase III clinical trials (16). Furthermore, it was awarded Rare Paediatric Disease Designation by the FDA in June 2020 (6). Therefore, this drug has promising clinical potential in the treatment of PH1. There are few studies on drugs targeted for HYPDH by iRNA. Only Xingsheng Li et al. reported that HYPDH-siRNA could reduce urine oxalate synthesis in mice models of PH1 (28). The efficacy and safety of the target need to be confirmed by more animal models and clinical trials.

In addition to the targeted therapies described above, new treatments also include oxalate removal, which reduces the levels of plasma and urine oxalate by reducing the absorption of intestinal oxalate (6), including probiotics (such as Oxalobacter formigenes) and oral enzymes (such as Alln-177) administration (4). However, neither of them was in phase III clinical trials, nor the efficacy has not yet been determined (19, 29). Among the above-mentioned therapeutic methods, the substrate reduction method based on iRNA targeting strategy is the most mature in clinical practice. Lumasiran, which targets GO, and Nedosiran, which targets LDHA, have the greatest body of evidence in clinical trials so far. In this paper, we focus on lumasiran.

## Mechanism action of lumasiran

According to the pathogenesis of PH1 described above, glycolate produced by body metabolism is converted into glyoxylate, the direct precursor of oxalate, by GO in liver peroxisome. Therefore, one of the ways to treat PH1 is to reduce the expression of GO. At present, there are two methods that can target GO, one is enzyme active site inhibition, the second is RNA interference. Whereas none of the candidate molecules for enzyme active site inhibition have entered clinical trials, lumasiran, a drug prepared by RNAi therapy, has proven to be clinically effective (30). In general, lumasiran, developed by Alnylam Pharmaceuticals, inhibits oxalate synthesis by silencing the expression of the GO-encoding HAO1 gene with RNAi therapy, thereby reducing oxalate levels in urine and plasma (9).

Lumasiran is a synthetic double-stranded small interfering RNA (siRNA) covalently conjugated with the N-acetylgalactosamine (GalNAc) (30, 31). Its ligand, GalNAc, a carbohydrate with high affinity for asialoglycoprotein receptor (ASGPR), which has a targeting effect, trigger endocytosis by binding to ASGPR, that delivering the siRNA preferentially targeted to the liver cytoplasm and adding into the RNA-induced silencing complex (30). In this complex, the antisense strand of lumasiran binds to the complementary sequence of the HAO1 mRNA, leading to its cleavage (9, 30). The lack of intact HAO1 mRNA, which involved in the encoding of GO, inhibits oxalate production. Due to GO being upstream of AGT, the deficient of which leads to PH1, the mechanism of action of lumasiran is independent of the underlying AGXT gene mutation (32) (Figure 1).

## Pharmacokinetics of lumasiran

Due to the rapid targeted uptake of lumasiran by the liver, it is rapidly distributed to the liver once administered and causes a rapid decline in plasma lumasiran levels (30). According to pharmacokinetic studies, lumasiran exhibited linear to slightly nonlinear, time-dependent activity in plasma (9). The average time to reach a maximum plasma concentration is 4 h after administration, the plasma protein binding can exhibit moderate to high (77%–85%), and it can be detected up to 24–48 h post dose (9, 30, 31). The average terminal plasma half-life is 5.2 h, the apparent central volume of distribution in adults is 4.9 L, and the plasma clearance is 26.5 L/h (9, 30, 31). Urinary clearance in both adults and children ranged from 2.0 to 3.4 L/h, with urinary excretion accounting for 7%–26% of the dose administered (9, 30, 31). Because lumasiran has a long half-life in liver and its pharmacokinetic characteristics in plasma, its administration frequency of once monthly or quarterly can still maintain the pharmacodynamic effect of the drug within the dosing interval without drug accumulation in plasma (9, 30). It is important to note that because of this, plasma concentrations do not reflect the extent or duration of lumasiran activity (9). Studies have shown that lumasiran is approved for use in all age groups (9), because of its stable pharmacokinetics which are not affected by age, race, sex, mild/moderate renal impairment, or mild/moderate liver impairment (30).

## Dosing regimen of lumasiran

Lumasiran is routinely administered subcutaneously, according to the FDA specification for lumasiran approved in November 2020. The administration regimen is determined based on body weight. The loading dose was 6 mg/kg once monthly for 3 consecutive months and the maintenance dose was 3 mg/kg once monthly for those less than 10 kg. For those patients who was 10 kg to less than 20 kg, the loading dose was also 6 mg/kg once monthly for 3 consecutive months and 6 mg/kg once every 3 months for the maintenance dose. While for those 20 kg and above, the loading dose was 3 mg/kg once monthly for 3 consecutive months and the maintenance dose was 3 mg/kg once every 3 months. No dose adjustment is necessary in patients with an eGFR between 30 and 90 ml/min/1.73 m^2^. In the FDA specification, lumasiran should be cautiously administered and closely monitored in patients with an eGFR <30 ml/min/1.73 m^2^, on dialysis and/or under 1 year of age, although dose adjustment is not mentioned. According to the ILLUMINATE-C clinical trial, which has completed the preliminary analysis period of 6 months, the safety and efficacy of lumasiran in the above patients can be confirmed, and the dose does not need to be adjusted. However, the extension of the trial has not yet been completed, and the use of the drug in these patients remains to be seen.

## Clinical evidence and evaluation of lumasiran

### Registered preclinical and clinical trials involving lumasiran

Preclinical and clinical trials of lumasiran comes from 5 studies, one of which is completed, and the others are currently active. The first phase I/II study of Lumasiran was initiated in 2016 and currently completed, which is a first-in-human, randomized, single-blind, placebo-controlled trial (NCT02706886) to evaluate the safety, pharmacokinetic, and pharmacodynamic of lumasiran in healthy adult participants and patients with PH1 (33). The study was divided into two parts: In Part A, 32 healthy adults were randomly assigned at 3:1 to lumasiran or placebo accepted ascending doses (0.3, 1, 3 or 6 mg/kg); In part B, 20 children (>6 years of age) and adults with PH1 were also randomly assigned at 3:1 to lumasiran or placebo receiving the doses of 1 mg/kg monthly, 3 mg/kg monthly, or 3 mg/kg quarterly (33). The results showed a 66% reduction in 24-h urinary oxalate excretion from baseline (1.69 mmol/24 h/1.7 3 m^2^) with a maximum reduction of 75% in the lumasiran group in part B. All patients achieved 24-h urinary oxalate levels ≤1.5 times the upper limit of normal (≤0.46 mmol/24 h/1.73 m^2^) (33). In terms of adverse events, most cases were mild to moderate in severity, including abdominal pain (18%), headache (18%), rhinitis (12%), nephrolithiasis (12%), and cough (12%). There were no severe adverse events in the lumasiran group, and two patients had adverse events related to lumasiran which consisted of mild or moderate transient injection-site reactions. Therefore, lumasiran has an acceptable safety profile (33).

At the end of the phase I/II study, all 20 patients in Part B were re-enrolled in a new study (NCT03350451), which is considered to be a long-term, open-label extension Phase 2 trials for continued dosing of 3 mg/kg quarterly with an estimated completion date of June 2023 (31). At a data cut-off March 1 2021 (median exposure 28 months) showed at Month 24 from baseline a 67.5% decrease in 24-h urinary oxalate excretion, a 72.2% decrease in 24-h urinary oxalate creatinine ratio, and a 51.0% decrease in plasma oxalate levels. In all patients, the 24 h urinary oxalate levels were less than 1.5 times the upper limit of normal (34). The most common lumasiran-related adverse events reported were injection site reactions, all of which were mild (34). Thus, consistent with the results of previous trials, lumasiran showed good efficacy and safety in children (>6 years) and adults.

There are currently three ongoing phase III trials. ILLUMINATE-A is an ongoing, randomized, double-blind, placebo-controlled phase III trial (NCT03681184) involving 39 PH1 patients (>6 years of age with an eGFR ≥ 30 ml/min/1.73 m^2^) who were randomly assigned at 2:1 to lumasiran or placebo where subjects were given 3 mg/kg once monthly for three doses, followed by maintenance doses given once every 3 months beginning 1 month after the last loading dose (35). The main analysis period was 6 months, and then it entered an extended period (up to 54 months) with lumasiran recipients continuing treatment with lumasiran 3 mg/kg quarterly and placebo recipients switching to lumasiran treatment with a loading dose of 3 mg/kg once monthly for 3 months and a maintenance dose of 3 mg/kg quarterly (35, 36). The analysis of trial data for the 6-month primary analysis period has been completed. It shows that the least-squares mean (LSM) difference between the lumasiran and placebo group for the change in 24-h urinary oxalate excretion was −53.5% (*P* < 0.001), with a reduction in the lumasiran group of 65.4% and an effect seen as early as month 1 (35). At Month 6, the 24 h urinary oxalate levels were less than 1.5 times the upper limit of normal in 84% of lumasiran recipients who received lumasiran (35). The difference in the percent change in the plasma oxalate level (lumasiran minus placebo) was −39.5% (*P* < 0.001) (35). Adverse events were also mild, transient injection-site reactions (35). The longest observation period reported in this trial was 24 months (in extended period), and the clinical results were also encouraging, with changes in 24-h urinary oxalate excretion, 24-h urinary oxalate levels, and plasma oxalate levels consistently decreasing from baseline without significant serious adverse events (36). It should be noticed that a limitation of this trial was that patients younger than 6 years of age or with an eGFR of less than 30 ml/min/1.73 m^2^ were not included. The trials that including the above two groups of patients were ILLUMINATE-B and ILLUMINATE-C, respectively.

ILLUMINATE-B (NCT03905694) is an ongoing, single-arm, open-label, multinational phase III trial of patients aged <6 years with PH1 (*N* = 18), with an eGFR >45 ml/min/1.73 m^2^ for those ≥12 months of age or a normal serum creatinine for patients <12 months of age (37). Similar to the ILLUMINATE-A trial, a 6-month preliminary analysis of the ILLUMINATE-B trial has been completed, while a 54-month long-term extension period is ongoing. The dosing regimen used in the trial was the standard drug dose described above, with load and maintenance doses based on body weight. The least-squares mean reduction from baseline in spot urinary oxalate: creatinine ratio was 72.0%, and the least-squares mean reduction in plasma oxalate was 31.7%, according to the analysis of the results of the 6-month completed trial (37). The most common adverse events were transient, mild, injection-site reactions (37). Therefore, the efficacy and tolerability profile of Lumasiran in children aged <6 years in this study were consistent with those reported in the ILLUMINATE-A trial (37). The data of the 12-month extended trial of ILLUMINATE-B have been reported. The spot urinary oxalate:creatinine ratio was maintained at 72% at month 12. The mean reduction from baseline in plasma oxalate level was improved to 47% at month 12. The most common adverse event was still proved to be a mild, transient injection site reaction.The results of the more than 12 months extended trial have not been reported (38). The trial lacked a placebo control group due to the limited number of patients aged <6 years with a diagnosed PH1 (37). Similarly, the absence of PH1 patients with abnormal renal function was a limitation of this study.

Evaluation of the efficacy and safety of Lumasiran in PH1 patients of all ages with impaired kidney function was performed in the ILLUMINATE-C trial (NCT0452200), which was a multicenter, multinational, single-arm, open-label Phase 3 study enrolled 21 patients of all ages with PH1, eGFR ≤ 45 ml/min/1.73 m^2^ (if age ≥12 months) or elevated serum creatinine (if age <12 months), and POx ≥ 20 µmol/L at screening, including patients with or without systemic oxalosis (39). The trial consisted of a 6-month primary analysis period and a long-term (54 months) extension period and was divided into two cohorts, in which Cohort A consisting of six patients who were not on hemodialysis at the time of study enrollment while Cohort B consisting of 15 patients who were receiving hemodialysis at the time of study enrollment (39). Data from the completed 6-month preliminary analysis period showed significant reductions in plasma oxalate (POx) in both cohorts, with least-squares mean reduction in POx of 33.3% in Cohort A and 42.4% in Cohort B from baseline (39). Studies have shown that the most common adverse events continue to be mild, transient injection-site reactions (39). Therefore, the efficacy and safety of lumasiran are acceptable. POx mean % reduction from baseline at month 12 was 69.3% and 34.3% in Cohorts A and B, respectively. POx AUC_0–24 h_ mean % reduction from baseline between hemodialysis sessions was 40.9% at month 12 (Cohort B). It should be mentioned that at 12 months of observation, two patients in Cohorts A started hemodialysis. In Cohorts B, one patient received kidney transplantation, stopped hemodialysis and continued lumasiran treatment; one patient received a liver/kidney transplant and stopped lumasiran treatment.The most common adverse events were the same as at 6 months. Combined with partial trial data that have been completed, Lumasiran can continuously reduce POx in PH1 patients with CKD 3B-5, which has been proved to be effective and safe (40).

ILLUMINATE-A, ILLUMINATE-B, and ILLUMINATE-C are ongoing which were expected to finish in January 2024, August 2024, and July 2025, respectively. Lumasiran has a good efficacy and safety in PH1 patients of all ages and with or without impaired kidney function, based on the completed data of three phase III clinical trials, namely, ILLUMINATE-A、ILLUMINATE-B and ILLUMINATE-C. It also provides reference evidence for clinical application of lumasiran. In addition, BONAPH1DE (NCT04982393) is a prospective observational study, currently recruiting, that aims to describe the natural course of patients with PH1 and to evaluate the long-term efficacy and safety of lumasiran (31).

### Case studies

PH1 is a rare disease with a wide range of clinical manifestations, some of which are mild or asymptomatic, and even ESRD being their first presentation (7). So, it is rarely diagnosed early. Thus, the number of patients younger than 6 years of age who were enrolled in the above trials, especially those younger than 1 year of age when they consented to the trials, was even smaller, with only 3 patients in ILLUMINATE-B, of which the minimum age was 3 months (37). To date, the youngest case report of lumasiran treatment for PH1 has been published at 9 days of age (41), and one case of twin male infants receiving lumasiran at 12 months of age has been reported (42).

Marie et al. reported the effect of lumasiran for PH1 in small infants (41). The first child was antenatally diagnosed of PH1 because his older sister was diagnosed with CKD stage 5 at 4 months of age, and UOx/creat and POx levels elevated immediately after birth (41). The first subcutaneous injection of lumasiran was administered at the day 9, and hyperhydration and potassium citrate were performed from month 2 (41). Although well tolerated, the outcome was unsatisfactory: the patient was found to have grade III nephrocalcinosis after 2 months of lumasiran treatment (41). Lumasiran treatment was therefore continued, but the dose was adjusted to 6 mg/kg/month with the addition of off-label stiripentol for only 2 months (41). After the seventh injection, UOx/creat decreased to normal (41). After the ninth injection, the dose was reduced to 3 mg/kg, while UOx/creat remaining stable (41). After 10 months of follow-up, kidney function remained normal and nephrocalcinosis was relieved to some extent (41). The other two patients, aged 2.5 months and 3.5 months, had significant clinical manifestations, and started lumasiran therapy shortly after diagnosis (41). Both patients had a marked decrease in UOx/creat and POx, and the latter had an improvement in nephrocalcinosis, but the former did not (41). No specific adverse events were observed in these three infants (41). This case report provides a reference for the treatment of small infants with PH1, because of lumasiran having a good efficacy and safety. Early diagnosis and intervention are very important for PH1 patients. Due to the short follow-up time and the first patient also received Stiripentol, the efficacy and safety of lumasiran still need to be observed.

Khaled et al. reported lumasiran treatment of PH1 in dizygotic twin gestation males delivered at 31 weeks of gestation, whose father had a 12-year history of kidney stones (42). Twin A was treated for kidney stone obstruction at 9 months of age, and still had recurrent kidney stone symptoms and urinary tract infection despite surgical intervention (42). After genetic analysis confirmed PH1, he was started on potassium citrate-citrate acid solution (Polycitra K) and pyridoxine at 11 months of age and was on lumasiran with the standard therapeutic dose at 12 months of age, while potassium citrate-citrate acid solution and pyridoxine were discontinued (42). Twin B was found to have nephrocalcinosis at 10 months of age and was treated with Polycitra K and pyridoxine before the gene confirmation of Twin A were available, and lumasiran injection was also started at 12 months of age (42). During the 8-month follow-up, there was no obvious evidence of disease recurrence and serious adverse events (42). So far this is the first report of PH1 on twins and also the first report of who were treated with lumasiran (42). This case report also suggests the clinical practice basis for the efficacy and safety of lumasiran in the treatment of young children with PH1.

For the use of lumasiran in patients outside of clinical trials, in addition to infants, cases have been reported in child who was confirmed PH1 after kidney transplant. The child whose mother having a history of polycystic kidney disease was found to have renal insufficiency when he was 5 years old, the cause of which was unknown and was thought to have renal dysplasia (43). He received a living-unrelated donor kidney transplant at age 7 years (43). The creatinine level increased on post-operative day 2 although surgical intervention. Hemodialysis was started on postoperative day 8 (43). Considering systemic oxalosis as the cause of transplant dysfunction, a genetic testing was sent and PH1 was confirmed (43). Lumasiran was started on post-operative day 34 and hemodialysis was discontinued gradually at approximately 4 months after transplantation (43). The decrease in urine oxalate levels during the first 5 months after initiation of lumasiran was consistent with that observed in clinical trials, he was able to maintain kidney function due to aggressive renal replacement therapy as well as the initiation of new targeted therapies for the disease (43). This demonstrates the efficacy and safety of luamsiran in the treatment of patients requiring hemodialysis after kidney transplant.

## Discussion

Primary hyperoxaluria type 1 is a rare recessive autosomal disorder caused by alanine-glyoxylate-aminotransferase (AGT) deficiency and is the most common and severe of three types of primary hyperoxaluria (1, 5). The accumulation of excess oxalate exceeds the threshold for renal excretion, which accumulates in the kidney and gradually in other organs, eventually leading to systemic oxalosis (44). Thus, PH1 is a devastating disease for kidney that can rapidly progress to ESRD in severe cases. An effective treatment for PH1, a disease with defective liver metabolism, is liver transplantation that is associated with morbidity and mortality (11). For patients with ESRD, liver–kidney transplantation is required, which increases the risk of morbidity and mortality. Before the introduction of new therapeutic agents, PH1 was treated with conservative regimens including intensive water intake, prescription of vitamin B6 and oral crystallization inhibitors, but it was not effective in preventing the occurrence of ESRD (44). Lumasiran, approved by the European Union and the United States in November 2020 for the treatment of PH1 patients of all ages, is a liver-directed RNA interference therapy, targeting the messenger RNA of glycolate oxidase (44), which can reduce the production of hepatic oxalate to achieve the purpose of treatment.

The high efficacy of lumasiran in the treatment of PH1 is evident from the five clinical trials described above and the related case reports. But there are still some things that clinicians should be aware of. First, these clinical trials demonstrating the efficacy of lumasiran have not yet been completed, and the longest follow-up for data analyses reported has been less than 24 months. In the case reports that have been published, the efficacy of lumasiran has been observed for even shorter periods, being a challenge to whether lumasiran is effective in the long-term treatment of PH1 disease. Second, in all clinical trials and published case reports, lumasiran was administered on top of conservative treatment (high water intake, potassium citrate with or without pyridoxine). Therefore, if lumasiran has a good curative effect, whether the conservative treatment can be stopped, and whether this will have an impact on the therapeutic effect of lumasiran in long term follow-up. Third, considering the mechanism action of lumasiran and its excellent performance in clinical trials and case reports, it is worth studying whether double liver/kidney transplantation can be replaced by isolated kidney transplantation and RNAi therapies in the future treatment strategy for PH1 (45, 46). This approach is intended to reduce the complications and mortality of liver transplantation, and complications of long-term immunosuppressive therapies (46). Anne-Laure Sellier-Leclerc et al. reported five PH1 patients of isolated kidney transplantation in combination with lumasiran, all of whom showed no signs of recurrent oxalate nephropathy after at least 6 months of follow-up (46). This seems to prove the feasibility of the scheme. However, Nizar et al. reported a case of recurrence of early oxalate nephropathy after kidney transplantation in a PH1 patient treated with lumasiran (47), which making the effectiveness of this approach questionable.

Compared with the efficacy of lumasiran, the safety of lumasiran needs more attention. Through clinical trials and related case reports, adverse events were concentrated on mild injection-site reactions, and no serious adverse events were identified. Likewise, the long-term safety of lumasiran remains to be seen, as the observation period of each clinical trial remaining short. In addition, the mechanism of action of lumasiran also poses certain challenges to the safety of this drug. Since lumasiran inhibits oxalate synthesis by silencing the expression of HAO1 gene encoding GO by RNAi therapy, whether long-term silencing of this gene will have related consequences is unknown. However, in rare cases of HAO1 biallelic loss of function variants (30), no obvious disease phenotype was found, which provided a certain basis for the safety of lumasiran. In addition, for patients with PH1, the biochemical hallmark is glycolic aciduria and together with hyperoxaluria. According to the pathogenesis of PH1, inhibition of GO results in the failure of glycolic acid in peroxisome to convert to glyoxalic acid, resulting in increased plasma glycolic acid levels (31). Further studies are needed to evaluate the effects of this glycolic acid buildup, for example, as glycolate is an acid (glycolic acid), whether it leads to metabolic acidosis needs to be explored.

The last thing to mention is the cost of lumasiran. Lumasiran’s specifications are 94.5 mg/0.5 ml each and the unit price is $67,980.00. Depending on the dosage, the annual cost of treatment can be up to $340,000.00 for patients less than 10 kg, up to $540,000.00 for patients between 10 and 20 kg, and more expensive for patients over 20 kg. The discounted price of the drug averages about $379,100 a year, according to an email from an Alnylam spokesperson (48). Its high price will affect its clinical popularity.

## Conclusion

Primary hyperoxaluria type 1 is a rare, severe disease caused by a deficiency of the alanine-glyoxylate-aminotransferase (AGT). Early diagnosis of PH1 is necessary. So far, liver transplantation is an effective treatment for the disease. In addition to conventional conservative treatment strategies, targeted therapy for PH1 has great potential. Among them, RNA interference therapy targeting hepatic GO (Lumasiran) and LDHa (Nedosiran) has the strongest evidence (21). Lumasiran, which was approved for use in 2020, has five ongoing clinical trials and, to date, has shown excellent efficacy with few adverse events. Although there are still some unsolved problems for lumasiran, its efficacy and safety are undeniable, and it provides a new direction and hope for the treatment of PH1.

## References

[1] Kletzmayr A Ivarsson ME Leroux JC. Investigational therapies for primary hyperoxaluria. Bioconjug Chem. (2020) 31:1696–707. 10.1021/acs.bioconjchem.0c00268 32539351

[2] Hopp K Cogal AG Bergstralh EJ Seide BM Olson JB Meek AM Phenotype-genotype correlations and estimated carrier frequencies of primary hyperoxaluria. J Am Soc Nephrol. (2015) 26:2559–70. 10.1681/ASN.2014070698 25644115 PMC4587693

[3] Dindo M Conter C Oppici E Ceccarelli V Marinucci L Cellini B. Molecular basis of primary hyperoxaluria: clues to innovative treatments. Urolithiasis. (2019) 47:67–78. 10.1007/s00240-018-1089-z 30430197

[4] Weigert A Martin-Higueras C Hoppe B. Novel therapeutic approaches in primary hyperoxaluria. Expert Opin Emerg Drugs. (2018) 23:349–57. 10.1080/14728214.2018.1552940 30540923

[5] Cochat P Ingelfinger JR Rumsby G. Primary hyperoxaluria. N Engl J Med. (2013) 369:649–58. 10.1056/NEJMra1301564 23944302

[6] Shee K Stoller ML. Perspectives in primary hyperoxaluria - historical, current and future clinical interventions. Nat Rev Urol. (2022) 19:137–46. 10.1038/s41585-021-00543-4 34880452 PMC8652378

[7] Soliman NA Nabhan MM Abdelrahman SM Abdelaziz H Helmy R Ghanim K Clinical spectrum of primary hyperoxaluria type 1: experience of a tertiary center. Nephrol Ther. (2017) 13:176–82. 10.1016/j.nephro.2016.08.002 28161266 PMC5921832

[8] Gupta A Somers MJG Baum MA. Treatment of primary hyperoxaluria type 1. Clin Kidney J. (2022) 15:i9–i13. 10.1093/ckj/sfab232 35592620 PMC9113429

[9] Scott LJ Keam SJ. Lumasiran: first approval. Drugs. (2021) 81:277–82. 10.1007/s40265-020-01463-0 33405070

[10] Hoppe B. Evidence of true genotype-phenotype correlation in primary hyperoxaluria type 1. Kidney Int. (2010) 77:383–5. 10.1038/ki.2009.471 20150937

[11] Devresse A Cochat P Godefroid N Kanaan N. Transplantation for primary hyperoxaluria type 1: designing new strategies in the era of promising therapeutic perspectives. Kidney Int Rep. (2020) 5:2136–45. 10.1016/j.ekir.2020.09.022 33305106 PMC7710835

[12] Du DF Li QQ Chen C Shi SM Zhao YY Jiang JP Updated genetic testing of primary hyperoxaluria type 1 in a Chinese population: results from a single center study and a systematic review. Curr Med Sci. (2018) 38:749–57. 10.1007/s11596-018-1941-y 30341509

[13] Cellini B. Treatment options in primary hyperoxaluria type I. Expert Opin Orphan Drugs. (2017) 5:309–19. 10.1080/21678707.2017.1298439

[14] Cochat P Hulton SA Acquaviva C Danpure CJ Daudon M De Marchi M Primary hyperoxaluria type 1: indications for screening and guidance for diagnosis and treatment. Nephrol Dial Transplant. (2012) 27:1729–36. 10.1093/ndt/gfs078 22547750

[15] Siener R Hoppe B Lohr P Muller SC Latz S. Metabolic profile and impact of diet in patients with primary hyperoxaluria. Int Urol Nephrol. (2018) 50:1583–9. 10.1007/s11255-018-1939-1 30039216

[16] Erger F Beck BB. A new era of treatment for primary hyperoxaluria type 1. Nat Rev Nephrol. (2021) 17:573–4. 10.1038/s41581-021-00449-9 34113016

[17] Hoppe B Martin-Higueras C. Improving treatment options for primary hyperoxaluria. Drugs. (2022) 82:1077–94. 10.1007/s40265-022-01735-x 35779234 PMC9329168

[18] Monico CG Olson JB Milliner DS. Implications of genotype and enzyme phenotype in pyridoxine response of patients with type I primary hyperoxaluria. Am J Nephrol. (2005) 25:183–8. 10.1159/000085411 15849466

[19] Rumsby G Hulton S-A. From pathogenesis to novel therapies in primary hyperoxaluria. Expert Opin Orphan Drugs. (2019) 7:57–66. 10.1080/21678707.2019.1571905

[20] Martin-Higueras C Luis-Lima S Salido E. Glycolate oxidase is a safe and efficient target for substrate reduction therapy in a mouse model of primary hyperoxaluria type I. Mol Ther. (2016) 24:719–25. 10.1038/mt.2015.224 26689264 PMC4886931

[21] Dejban P Lieske JC. New therapeutics for primary hyperoxaluria type 1. Curr Opin Nephrol Hypertens. (2022) 31:344–50. 10.1097/MNH.0000000000000790 35266883 PMC9232952

[22] Lai C Pursell N Gierut J Saxena U Zhou W Dills M Specific inhibition of hepatic lactate dehydrogenase reduces oxalate production in mouse models of primary hyperoxaluria. Mol Ther. (2018) 26:1983–95. 10.1016/j.ymthe.2018.05.016 29914758 PMC6094358

[23] Wood KD Holmes RP Erbe D Liebow A Fargue S Knight J. Reduction in urinary oxalate excretion in mouse models of Primary Hyperoxaluria by RNA interference inhibition of liver lactate dehydrogenase activity. Biochim Biophys Acta Mol Basis Dis. (2019) 1865:2203–9. 10.1016/j.bbadis.2019.04.017 31055082 PMC6613992

[24] Kempf C Pfau A Holle J Muller-Schluter K Bufler P Knauf F Stiripentol fails to lower plasma oxalate in a dialysis-dependent PH1 patient. Pediatr Nephrol. (2020) 35:1787–9. 10.1007/s00467-020-04585-5 32418144 PMC7385015

[25] Summitt CB Johnson LC Jonsson TJ Parsonage D Holmes RP Lowther WT. Proline dehydrogenase 2 (PRODH2) is a hydroxyproline dehydrogenase (HYPDH) and molecular target for treating primary hyperoxaluria. Biochem J. (2015) 466:273–81. 10.1042/BJ20141159 25697095 PMC4377293

[26] Liebow A Li X Racie T Hettinger J Bettencourt BR Najafian N An investigational RNAi therapeutic targeting glycolate oxidase reduces oxalate production in models of primary hyperoxaluria. J Am Soc Nephrol. (2017) 28:494–503. 10.1681/ASN.2016030338 27432743 PMC5280024

[27] Frishberg Y Zeharia A Lyakhovetsky R Bargal R Belostotsky R. Mutations in HAO1 encoding glycolate oxidase cause isolated glycolic aciduria. J Med Genet. (2014) 51:526–9. 10.1136/jmedgenet-2014-102529 24996905

[28] Li X Knight J Fargue S Buchalski B Guan Z Inscho EW Metabolism of (13)C5-hydroxyproline in mouse models of Primary Hyperoxaluria and its inhibition by RNAi therapeutics targeting liver glycolate oxidase and hydroxyproline dehydrogenase. Biochim Biophys Acta. (2016) 1862:233–9. 10.1016/j.bbadis.2015.12.001 26655602 PMC4706777

[29] Hatch M Freel RW. A human strain of Oxalobacter (HC-1) promotes enteric oxalate secretion in the small intestine of mice and reduces urinary oxalate excretion. Urolithiasis. (2013) 41:379–84. 10.1007/s00240-013-0601-8 23959075 PMC3815490

[30] Hulton S-A. Lumasiran: expanding the treatment options for patients with primary hyperoxaluria type 1. Expert Opin Orphan Drugs. (2021) 9:189–98. 10.1080/21678707.2021.2003779

[31] D’ambrosio V Ferraro PM. Lumasiran in the management of patients with primary hyperoxaluria type 1: from bench to bedside. Int J Nephrol Renovasc Dis. (2022) 15:197–206. 10.2147/IJNRD.S293682 35747094 PMC9211742

[32] Goga A Stoffel M. Therapeutic RNA-silencing oligonucleotides in metabolic diseases. Nat Rev Drug Discov. (2022) 21:417–39. 10.1038/s41573-022-00407-5 35210608

[33] Frishberg Y Deschenes G Groothoff JW Hulton SA Magen D Harambat J Phase 1/2 study of lumasiran for treatment of primary hyperoxaluria type 1: a placebo-controlled randomized clinical trial. Clin J Am Soc Nephrol. (2021) 16:1025–36. 10.2215/CJN.14730920 33985991 PMC8425611

[34] Magen D Groothoff JW Hulton S-A. Long-term treatment with lumasiran: results from the phase 2 open-label extension study. Kidney Int Rep. (2022) 7:S195. 10.1016/j.ekir.2022.01.465

[35] Garrelfs SF Frishberg Y Hulton SA Koren MJ O’riordan WD Cochat P Lumasiran, an RNAi therapeutic for primary hyperoxaluria type 1. N Engl J Med. (2021) 384:1216–26. 10.1056/NEJMoa2021712 33789010

[36] John L Jaap G Yaacov F. Efficacy and safety of lumasiran in patients with primary hyperoxaluria type 1: 24-month analysis of the ILLUMINATE-A trial. Am J Kidney Dis. (2022) 79:S1–2. 10.1053/j.ajkd.2022.01.009

[37] Sas DJ Magen D Hayes W Shasha-Lavsky H Michael M Schulte I Phase 3 trial of lumasiran for primary hyperoxaluria type 1: a new RNAi therapeutic in infants and young children. Genet Med. (2022) 24:654–62. 10.1016/j.gim.2021.10.024 34906487

[38] Hayes W Sas DJ Magen D Shasha-Lavsky H Michael M Sellier-Leclerc AL Efficacy and safety of lumasiran for infants and young children with primary hyperoxaluria type 1: 12-month analysis of the phase 3 ILLUMINATE-B trial. Pediatr Nephrol. (2022). 10.1007/s00467-022-05684-1 PMC9925547 35913563

[39] Michael M Groothoff JW Shasha-Lavsky H Lieske JC Frishberg Y Simkova E Lumasiran for advanced primary hyperoxaluria type 1: phase 3 ILLUMINATE-C trial. Am J Kidney Dis. (2022). 10.1053/j.ajkd.2022.05.012 35843439

[40] Frishberg Y. Lumasiran for patients with primary hyperoxaluria type 1 and impaired kidney function: 12-month analysis of the phase 3 ILLUMINATE-C trial. In: ASN Kidney week (2022).

[41] Meaux MN Sellier-Leclerc AL Acquaviva-Bourdain C Harambat J Allard L Bacchetta J. The effect of lumasiran therapy for primary hyperoxaluria type 1 in small infants. Pediatr Nephrol. (2022) 37:907–11. 10.1007/s00467-021-05393-1 35015123

[42] Aldabek K Grossman OK Al-Omar O Fox JA Moritz ML. Infantile primary hyperoxaluria type 1 treated with lumasiran in twin males. Cureus. (2022) 14:e21673. 10.7759/cureus.21673 35237473 PMC8882078

[43] Stone HK Vandenheuvel K Bondoc A Flores FX Hooper DK Varnell CD Jr. Primary hyperoxaluria diagnosed after kidney transplant: a review of the literature and case report of aggressive renal replacement therapy and lumasiran to prevent allograft loss. Am J Transplant. (2021) 21:4061–7. 10.1111/ajt.16762 34254430 PMC8639665

[44] Gillion V Dahan K Scohy A Devresse A Godefroid N. Lessons for the clinical nephrologist: lumasiran as the future cornerstone treatment for patients with primary hyperoxaluria type 1? J Nephrol. (2022). 10.1007/s40620-022-01435-5 35986861 PMC9998308

[45] Tandoi F. Combined liver kidney transplantation for primary hyperoxaluria type 1: will there still be a future? Current transplantation strategies and monocentric experience. Pediatr Transplant. (2021) 00:e14003. 10.1111/petr.14003 33742750

[46] Sellier-Leclerc AL Metry E Clave S Perrin P Acquaviva-Bourdain C Levi C Isolated kidney transplantation under lumasiran therapy in primary hyperoxaluria type 1: a report of 5 cases. Nephrol Dial Transplant. (2022). 10.1093/ndt/gfac295 36307929

[47] Joher N Moktefi A Grimbert P Pagot E Jouan N El Karoui K Early post-transplant recurrence of oxalate nephropathy in a patient with primary hyperoxaluria type 1, despite pretransplant lumasiran therapy. Kidney Int. (2022) 101:185–6. 10.1016/j.kint.2021.10.022 34991805

[48] Jaklevic MC. First drug approved for rare genetic disorder affecting kidneys. JAMA. (2021) 325:214. 10.1001/jama.2020.26382 33464323

